# Loneliness in Resettlement Among Multi-Ethnic Resettled Refugees in Ohio

**DOI:** 10.1007/s40615-025-02614-3

**Published:** 2025-09-30

**Authors:** Saruna Ghimire, Isha Karmacharya, Aman Shrestha, Ahmed Danquah, Shuayb Jet Jama, Seleshi Asfaw, Surendra Bir Adhikari

**Affiliations:** 1 Department of Sociology and Gerontology and Scripps Gerontology Center, Miami University, Oxford, OH 45056 USA; 2 Doctoral Program in Gerontology, University of Maryland, Baltimore (UMB) & Baltimore County (UMBC), Baltimore, MD 21201 & 21250 USA; 3 ETSS Tewahedo Social Services, 4300 E. Broad St, Suite D, Columbus, OH 43213 USA; 4 Ohio Department of Mental Health & Addiction Services, 30 E. Broad St, Columbus, OH 43215 USA

**Keywords:** Loneliness, Social isolation, Refugees, Mental health, Social support, Resilience

## Abstract

**Background:**

This study assessed the prevalence and factors associated with loneliness among five resettled communities in Ohio: Afghan, Bhutanese, Congolese, Ethiopi an/Eritrean, and Somali.

**Methods:**

A cross-sectional online survey of 572 participants was conducted with the help of local community organizations. Loneliness was measured using the three-item UCLA Loneliness Scale and categorized into two groups: “not lonely” and “lonely.” The final analytic sample comprised 458 participants. Binary logistic regression was used to analyze key predictors, including healthcare access, mental and physical health, social support, and resilience.

**Results:**

Of the participants, 29% reported loneliness. Participants with regular access to a doctor were 56% less likely to experience loneliness than those without access [adjusted odds ratio (aOR) = 0.44, 95% CI: 0.29–0.69], while fair/poor self-rated health tripled the experience (aOR = 3.07, 95% CI: 1.42–6.63). Mental health was the strongest predictor, with anxiety increasing the odds of loneliness by over eight times (aOR = 8.43, 95% CI: 4.46–15.93) and depression by more than three times (aOR = 3.53, 95% CI: 1.99–6.26). Experiencing racial discrimination increased the odds of loneliness by 73% (aOR = 1.73, 95% CI: 1.10–2.74). Low resilience quintupled the odds of loneliness (aOR = 5.07, 95% CI: 2.79–9.20), while low social support doubled such odds (aOR = 2.50, 95% CI: 1.04–6.03).

**Conclusion:**

The study found a high prevalence of loneliness among adults in resettled communities, which underscores the need to address physical and mental health, healthcare access, and social support. Especially, it is critical to develop and implement culturally tailored interventions to reduce loneliness and improve the well-being of these communities.

**Supplementary Information:**

The online version contains supplementary material available at https://doi.org/10.1007/s40615-025-02614-3.

## Introduction

### Loneliness: A Public Health Concern

Loneliness, defined as the subjective feeling of isolation, disconnection, or lack of meaningful social interaction, occurs when there is a gap between the social connections individuals desire and those they actually experience [1]. It is a profound and multifaceted issue that extends far beyond a fleeting emotional state and has increasingly been recognized as a critical public health concern with wide-reaching consequences [2]. The health risks associated with loneliness are comparable to, or even greater than, those posed by well-known public health threats such as smoking, obesity, and air pollution [3]. Loneliness has been linked to an increased risk of mental health disorders, a 30% higher risk of developing heart disease and stroke, and a 50% increased risk of dementia [4]. The multifaceted negative impacts of loneliness on health, considered more detrimental than those of obesity or physical inactivity [3], create a ripple effect that can lead to premature death. Furthermore, the mortality impact of loneliness has been compared to smoking 15 cigarettes a day [2]. The recent declaration by the U.S. Surgeon General, labeling loneliness an epidemic [2], underscores the gravity of this issue and highlights the urgent need for coordinated societal and healthcare responses. Loneliness, therefore, is not just a personal experience—it is a societal issue with substantial public health implications, including increased healthcare costs, reduced workforce productivity, and greater social fragmentation [2, 3].

Although loneliness affects individuals across all populations, its causes and consequences may be particularly complex and pronounced among resettled refugee groups, who face unique sociocultural and structural barriers to social connection.

### Resettled Refugees in the U.S.: Vulnerabilities to Loneliness

Loneliness is particularly pronounced among immigrants and refugees compared to native citizens [5]. Since the enactment of the Refugee Act in 1980, the United States has provided refuge to over 3.8 million individuals categorized as refugees and asylees [6]. As of fiscal year (FY) 2024, the number of refugees admitted into the United States was 100,034, which was about a 67% increase from the number in FY 2023 [7]. In recent years, Ohio has emerged as a significant resettlement destination, welcoming approximately 2,818 new refugee in 2023 and 4,312 in 2024 [8]. These newcomers have settled across the state, bringing both opportunities and challenges to Ohio’s broader communities. This influx necessitates a deeper understanding of the refugee experience and the factors that influence successful integration.

Studies focusing on specific refugee populations have identified a notable overlap in factors contributing to loneliness, indicating shared challenges across diverse communities [9]. Resettled refugees face pervasive challenges like isolation and marginalization, forced migration, trauma, loss of social networks, language barriers, and cultural dislocation, which can significantly hinder their well-being and acculturation into host communities [10]. The separation from familiar environments and loved ones, coupled with the need to rebuild their lives in foreign settings, further deepens their sense of isolation [9]. For refugees from collectivist cultures—like Afghanistan, Bhutan, and Somalia—being apart from family can be particularly distressing. It often exacerbates feelings of loneliness by disrupting core social and emotional bonds central to their sense of belonging [9]. Among Bhutanese refugees, disruptions in family relationships, challenges in adapting to new communities, safety concerns, and communication barriers to expressing emotional distress have been identified as key risk factors contributing to a high prevalence of suicide [11, 12]. Similarly, Afghan refugees have reported loneliness stemming from separation from family and cultural disconnection, accompanied by a lower likelihood of seeking help despite experiencing higher rates of mental health issues [13, 14]. In addition, many refugees face social exclusion, racial discrimination, and barriers to accessing essential services. These challenges increase their vulnerability to loneliness and hinder their ability to fully integrate into host communities [15].

### Gaps in Current Literature

Despite the growing recognition of loneliness as a public health concern, significant gaps remain in understanding how this issue affects resettled communities, particularly within the United States. While international studies have highlighted elevated levels of loneliness among resettled populations compared to host communities, U.S.-based research has lagged behind in providing comprehensive, population-level data. For example, a 17-year study of the U.S. population (2003–2020) revealed a notable overall increase in social isolation, especially among low-income and older adults; and that household companionship was declining as face-to-face interactions with friends and family were increasingly replaced by digital communication [16].

Most recent international studies, specifically from Europe, on loneliness in the resettled demographics consistently show resettled populations experiencing higher levels of loneliness compared to host populations. One such research in Germany found that most resettled adults were socially isolated and experienced moderate loneliness, even with some social support [17]. A comparative study across Europe also highlighted that individuals with a migration background are more prone to loneliness than non-migrants, with first-generation immigrants arriving after age 18 being particularly vulnerable [18]. In the UK, 58% of refugees and migrants identified loneliness as their greatest challenge when trying to settle in a new country [19].

A recent systematic review on loneliness and social isolation among refugees resettled in high-income countries found that the prevalence of social isolation ranged from 9.8% to 61.2%, while loneliness ranged from 15.9% to 47.7%—notably higher than rates seen in the general population [9]. The review included several studies from the United States, most of which primarily used qualitative methods, with a few utilizing quantitative or mixed methods, and were mostly conducted before or during the early 2000s. These studies focused on refugees from regions such as the Middle East (Iraq, Syria, Afghanistan), Africa (Somalia, Sudan, Congo, Ethiopia), Southeast Asia (Vietnam, Cambodia, Laos), Eastern Europe (Bosnia), and Central America (El Salvador). The prevalence of loneliness and isolation varied significantly, with figures ranging from 19.7% to 60% [9]. Common risk factors included family separation, language barriers, discrimination, and cultural differences. While these studies offer valuable insights, the high reliance on qualitative methods limits the ability to assess prevalence and correlates on a population level. The wide range of reported prevalence of loneliness (15.9% to 47.7%) specifically indicates the need for more precise and representative estimates [9].

To summarize, despite growing recognition of loneliness as a public health crisis in the United States [2], research on this issue among resettled refugees remains limited in scope and relevance. Most existing U.S.-based studies have relied primarily on qualitative methods or were conducted prior to 2000, making it difficult to quantify current prevalence or identify key predictors of loneliness at the population level. Furthermore, there is a notable gap in understanding how loneliness affects refugees in non-traditional resettlement states like Ohio, where refugee demographics have shifted significantly in recent years. This study addresses these gaps by providing current, quantitative data on the prevalence and correlates of loneliness among five multi-ethnic refugee communities in Ohio. In doing so, it offers timely and new evidence to inform targeted public health responses and culturally appropriate interventions.

### Theoretical Framework

The Social Ecological Model (SEM) served as the theoretical framework for our study, offering a comprehensive lens to understand how health outcomes, specifically loneliness, are shaped by dynamic interactions across individual, interpersonal, community, and societal levels [20, 21]. By moving beyond a purely individualistic explanation, SEM allows us to situate loneliness within the broader social and structural contexts that resettled refugees navigate. This framework informed both the selection and organization of our predictor variables, with each level of the model corresponding to specific domains of influence. At the individual level, we examine personal health and psychological characteristics—such as self-rated health, chronic disease status, anxiety, depression, and resilience—which reflect the internal resources and vulnerabilities that refugees carry from their pre-migration, transit, and resettlement experiences. The interpersonal level is captured through perceived social support, acknowledging the central role of disrupted or intact social networks in buffering against isolation. At the community level, we include healthcare access indicators to assess the availability of institutional resources that support social integration and a sense of belonging. Finally, the societal level is represented by perceived racial discrimination, which captures the influence of macro-level forces such as structural inequality and cultural exclusion that undermine refugees’ sense of belonging in host communities. The relevance and utility of SEM in refugee health research are well-supported in the literature. Two comprehensive reviews illustrate this application in the literature among the refugee population [22, 23]. A systematic review of 63 studies on the mental health of refugee children and adolescents identified risk and protective factors at every level of the SEM [22], including individual (e.g., trauma exposure), family (e.g., parental mental health, family cohesion), community (e.g., school connectedness, peer support), and sociocultural (e.g., discrimination, acculturation) influences. A second review on refugee women’s health also applied the SEM framework to organize its findings and highlighted multi-level challenges such as exposure to violence and high post-traumatic stress disorder (PTSD) rates, disrupted social support due to language barriers, and difficulties accessing care stemming from low health literacy, cultural differences, and limited system navigation [23]. Both reviews emphasized the importance of addressing influences at every ecological level and concluded that effective prevention and intervention strategies must be multi-level in nature. Grounded in this body of work, our use of SEM enables a more holistic and contextually grounded analysis of loneliness among resettled refugees, shedding light on how nested ecological factors collectively shape their mental health outcomes.

### Study Objectives and Hypotheses

This study seeks to fill the aforementioned gap by investigating the prevalence of loneliness among Ohio’s multi-ethnic resettled populations and identifying the key contributing factors. We hypothesized that loneliness would be more prevalent among participants with poor self-rated health, chronic illness, symptoms of depression or anxiety, experiences of racial discrimination, low resilience, and low perceived social support, and less prevalent among those with regular access to a healthcare provider.

As Ohio continues to welcome refugees and foster inclusive communities, addressing loneliness is essential to ensuring the well-being of these individuals and that they not only find safety but also experience a true sense of belonging. The findings from this research will help inform policies and interventions aimed at promoting social connectedness and reducing loneliness among the resettled refugees and guide the development of more effective support systems and structures across the state.

## Methods

### Study Design and Eligibility Criteria

This study employed an online cross-sectional survey to gather data for a comprehensive health needs assessment, conducted between November 2023 and February 2024. The Ethiopian Tewahedo Social Services (ETSS; https://ethiotss.org/), a prominent refugee and immigrant serving organization in Central Ohio since 2000, led the data collection process with support from local community-based organizations. These partnerships were crucial in reaching the target populations and ensuring the cultural and linguistic appropriateness of the survey materials.

Eligible participants met the following criteria: they were 18 years or older, had refugee backgrounds from one of five specific countries—Afghanistan, Bhutan, Congo, Ethiopia and/or Eritrea, or Somalia—had resettled in the United States, and were currently residing in Ohio. We intentionally use the term “resettled” instead of “refugee” to describe our study population, as most individuals are now either permanent residents or U.S. citizens. This terminology better reflects their current status and integration within the United States. These five communities were selected purposefully based on several factors. First, ETSS has strong relationships and commitments from local leaders in these communities, which facilitated access and data collection. Second, these countries represent some newly emerging resettled communities and contribute to the highest number of refugees resettled in Ohio, making them important groups for understanding refugee health disparities in the state [8].

### Sample Size and Sampling Technique

The required sample size for the study was determined using Cochran’s formula [24], which is widely used in survey research to calculate sample size for large populations. The sample size formula is: n = z^2^pq/d^2^, where n is the required sample size, z (= 1.96) is the z-score for a 95% confidence level, *p* (= 50% or 0.5) is the estimated population proportion, q is 1-*p* (= 0.5), and d is the desired level of precision (at 5%, or 0.05). Based on the calculation, the minimum sample size required was 385 participants. However, due to the successful outreach and engagement efforts, a total of 572 online responses were obtained.

To ensure broad participation, a variety of recruitment strategies were employed, tailored to each community’s communication preferences. Local community-based organizations representing each of the five resettled communities played a crucial role in disseminating survey information. Flyers containing a link to the online survey were distributed at community centers, religious sites such as churches and temples, as well as grocery stores and restaurants frequented by the target populations. Digital outreach was another key method, with ETSS staff and community leaders sending survey invitations via email, social media platforms like Facebook and Instagram, and text messages. In addition, community leaders conducted home visits to promote the survey and assisted those who needed help accessing or navigating the online platform. Furthermore, the community’s strong social networks were leveraged to spread information about the survey through word of mouth. Leaders, community members, and volunteers encouraged participation, explaining the significance of the study and its potential benefits for the community.

### Data Collection

The survey was self-administered using Qualtrics, an online survey platform, and was available only in English. Community leaders from each group reviewed the survey to ensure its cultural appropriateness and relevance. The preference was to offer the survey in multiple languages to ensure broader outreach. However, logistical constraints limited its availability to English, which presented a significant limitation.

The survey tool collected demographic information and covered various aspects of health needs, including access to healthcare services, health behaviors, barriers to care, social determinants of health, chronic diseases, and mental health. A detailed description of each variable used in the current study is provided below.

### Measures

The primary outcome variable in this study was loneliness, with key predictors including health-related and psychosocial variables. Health-related predictors included access to healthcare when needed, access to a regular doctor, self-rated health, chronic diseases, anxiety, and depression. Psychosocial predictors included perceived discrimination for race, resilience, and social support. Sociodemographic control variables, such as age, sex assigned at birth, marital status, education, current employment status, and the community they represented, were also considered.

#### Dependent Variable

##### Loneliness

It was measured using the 3-item UCLA Loneliness Scale, a validated tool designed to assess subjective feelings of loneliness and social isolation [25]. This scale is widely used due to its brevity and ease of administration, making it ideal for large-scale surveys and contexts where time or resources are limited [26], and has been used in studies including diverse populations and cross-cultural adaptation [27]. The UCLA Loneliness Scale has previously demonstrated excellent internal consistency, with Cronbach's alpha typically ranging from 0.89 to 0.94 across various studies, and test–retest reliability reported at r = 0.73 [25]. Its validity is also supported by strong correlations with other loneliness measures and indicators of interpersonal relationships and well-being [25]. For the current study, the internal consistency of this multidimensional construct, assessed using McDonald’s Omega [28], yielded a reliability coefficient of 0.84, indicating good reliability.

The 3-item scale assessed how often participants felt (a) a lack of companionship, (b) left out, and (c) isolated from others. Responses were rated on a 3-point Likert scale: 1 = “Hardly ever,” 2 = “Some of the time,” and 3 = “Often.” Scores for each item were summed to create a total score ranging from 3 to 9, with higher scores reflecting greater loneliness. Consistent with existing literature, the scores were dichotomized to define the outcome variable as a binary variable: scores between 3 and 5 were classified as “not lonely,” while scores from 6 to 9 were categorized as “lonely” [29].

#### Primary Independent Variables

##### Access to Healthcare when needed

To assess healthcare access, participants were asked, “In the past 12 months, was there a time when you needed to see a doctor but could not due to financial reasons?” Initially, the responses were in yes/no format, with those who answered “0. No” categorized as having “healthcare access” and those who answered “1. Yes” as having “no healthcare access.” To simplify the interpretation, we created a new variable"access to healthcare when needed"and reverse-coded the responses:"0. Yes"indicated having access to healthcare when needed, while"1. No"indicated could not access healthcare when needed.

##### Access to a Regular Doctor

Participants were asked, “Do you have a doctor who regularly treats you when you are sick?” Responses were recorded as “Yes” or “No.”

##### Self-Rated Health

Participants were asked to rate their overall health using the question, “How would you describe your health?” Response options ranged from “excellent” to “poor.” Due to low counts, the responses “fair” and “poor” were combined into a single category, labeled as “fair/poor health.” Self-rated health is a subjective and immediate assessment of overall health that has been validated in different ethnic groups and settings [30]. A prior study discovered that self-rated health was linked to more objective health indicators and remained an independent predictor of mortality, even after adjusting for the objective measures [31].

##### Chronic Disease Status

Participants were asked whether a healthcare professional had ever diagnosed them with any of the following chronic conditions: high blood pressure, high cholesterol, diabetes, heart attack, angina, coronary heart disease, stroke, asthma, chronic obstructive pulmonary disease (COPD), arthritis, kidney disease, or cancer. An open-ended question at the end of this section allowed participants to specify any other chronic conditions not listed. Responses for each condition were recorded in a binary format (1 ="Yes,"0 ="No"). The total number of chronic conditions was then summed, and participants were categorized into two groups: 0 =“No chronic condition” and 1 =“One or more chronic conditions.”

##### Depression and Anxiety

Symptoms of depression and anxiety were measured using the Patient Health Questionnaire-4 (PHQ-4), a concise, self-reported tool designed to assess mental health [32]. The PHQ-4 consists of two items: first two questions for anxiety and last two for depression. Participants were asked to reflect on their symptoms over the past seven days, responding to four key questions: (a) “How often have you had little interest or pleasure in doing things?”; (b) “How often have you felt down, depressed, or hopeless?”; (c) “How often have you felt nervous, anxious, or on edge?”; and (d) “How often have you found it hard to stop or control worrying?” Response options were "not at all,""several days,""more than half the days,"and"nearly every day,"with corresponding scores of 0, 1, 2, and 3, respectively. The total scores for the depression and anxiety subscales ranged from 0 to 6, with scores of 3 or higher on each subscale indicating a potential presence of depression or anxiety, respectively.

The PHQ-4 has been widely validated in both general and resettled populations, demonstrating strong reliability and structural consistency. In a nationally representative household survey, the confirmatory factor analyses showed a well-fitting two-factor model, with structural consistency across age and gender [32]. Further, the tool showed good construct validity through its correlations with other mental health measures and demographic risk factors for anxiety and depression [32]. In studies focusing on resettled populations, such as research involving Syrian refugees, the PHQ-4 has been found to exhibit strong internal consistency, with Cronbach’s alpha scores of 0.76 for depression and 0.83 for anxiety, and confirmatory factor analyses further indicating a good model fit [33]. In the present study, the PHQ-4 demonstrated robust reliability, with a McDonald’s Omega score of 0.83, further supporting its use in assessing mental health in diverse populations.

##### Perceived Discrimination for Race

Perceived discrimination was measured using the Everyday Discrimination Scale (EDS) [34], which consists of six items assessing experiences of daily discrimination. These experiences included being treated with less courtesy, receiving inferior service in restaurants or stores, being perceived as less intelligent, encountering people who seemed afraid of them, facing threats or harassment, or receiving suboptimal healthcare. If participants reported experiencing any form of discrimination, they were asked to identify the primary reason they believed it occurred. They could select from ten possible reasons (e.g., race, religion, language proficiency) or provide their own explanation for the perceived discrimination. For this study, perceived racial discrimination was measured as a binary variable (“yes” or “no”), depending on whether participants believed race was the reason for their experiences of discrimination.

The EDS is widely used across diverse populations and has demonstrated strong internal consistency, as well as robust convergent and divergent validity [35]. The tool has also been applied to resettled populations, including Syrian refugees in Germany, where it showed strong internal reliability with a Cronbach’s alpha of 0.90 [36]. In this study, the EDS also showed an acceptable internal consistency with a McDonald’s Omega reliability score of 0.7.

##### Resilience

In this study, resilience was measured using the widely recognized Connor-Davidson Resilience Scale (CD-RISC) [37]. The CD-RISC consists of 10 items, each rated on a 5-point Likert scale ranging from 0 ("Not true at all") to 4 ("True nearly all the time"). Participants were asked to reflect on their ability to manage various aspects of resilience over the past month, such as: (a) adapting to changes, (b) handling challenges that arise unexpectedly, (c) maintaining a sense of humor in the face of problems, (d) coping with stress in ways that strengthen them, (e) bouncing back from illness, injury, or other hardships, (f) achieving goals despite obstacles, (g) staying focused and thinking clearly under pressure, (h) resisting discouragement from failure, (i) perceiving themselves as strong when confronted with life’s difficulties, and (j) managing unpleasant or painful emotions like sadness, fear, or anger. To determine an individual's overall resilience level, the responses across the 10 items were summed, resulting in a cumulative score ranging from 0 to 40. A higher total score indicated a higher level of resilience. Given the non-normal distribution and skewness observed in the data, the total scores were grouped into three categories—low, medium, and high resilience—based on tertile distribution.

The CD-RISC has been widely used across diverse populations to measure psychological resilience [38]. A study among Syrian refugees in Istanbul, Turkey, demonstrated that CD-RISC has strong internal reliability, with a Cronbach's alpha of 0.90 [38]. Additionally, the scale has been validated as a robust psychometric tool for assessing resilience, as evidenced by its demonstrated discriminant and predictive validity [39]. In this study, the CD-RISC also exhibited excellent reliability, with a McDonald’s Omega coefficient of 0.95, further affirming its effectiveness as a measurement tool.

##### Social Support

In this study, social support was assessed using the Multidimensional Scale of Perceived Social Support (MSPSS), a well-established tool for measuring perceived support from family, friends, and significant others [40]. The MSPSS consists of 12 items, each rated on a 7-point Likert scale ranging from 1 ("very strongly disagree") to 7 ("very strongly agree"). Participants'overall social support score was calculated by summing their responses, yielding a possible score range of 12 to 84, with higher scores indicating greater perceived social support [40]. The mean score, derived by dividing the total score by 12, was categorized into"low"(1 to 2.9),"medium"(3 to 5), and"high"(5.1 to 7) levels of support [40].

The MSPSS has consistently shown strong psychometric properties across different populations. For example, studies involving migrants have demonstrated its high construct validity and excellent reliability, with a Cronbach’s alpha of 0.90 [41]. Similar results were observed in research on adult refugees, where the scale achieved a Cronbach’s alpha of 0.88 [38]. In this study, the MSPSS also exhibited excellent internal consistency, with a McDonald’s Omega coefficient of 0.95, further affirming its reliability as a measure of perceived social support.

#### Control Variables

Sociodemographic characteristics were included as control variables to adjust for their potential influence on the outcomes. Age was categorized into four groups: 18–24, 25–34, 35–44, and 45 years and above. Sex at birth was recorded as male or female. Marital status was grouped into two categories due to low counts in some subgroups: "married or in a relationship" and "never married, separated, divorced, or widowed. "Education level was classified into four categories:"college graduate,""some college,""high school graduate or equivalent," and "none or less than high school."Current employment status was noted as either "employed" or "not employed". Lastly, participants were categorized into one of the five resettled communities they represented: Afghanistan, Bhutan, Congo, Ethiopia and/or Eritrea, or Somalia.

### Data Analyses

Data from Qualtrics were imported into SAS version 9.4 [42] for all preprocessing and analyses. To ensure that multicollinearity was not an issue, both a correlation matrix and the variance inflation factor (VIF) were assessed, with VIF values below 2.5 indicating no concerns regarding multicollinearity. A jackknife diagnostic, an advanced technique used to evaluate model robustness and stability, identified one influential observation, which was removed using a case-wise deletion method. Only complete cases were included, resulting in a final analytical sample of 458 observations.

Since all variables in the analysis were categorical, descriptive statistics were reported as counts and percentages, and bivariate relationships between variables were assessed using Pearson's Chi-square test. To explore the relationships between the independent and dependent variables, binary logistic regression was conducted. Both unadjusted and adjusted logistic regression models were developed, with odds ratios and 95% confidence intervals (CI) reported to quantify the odds of loneliness across different levels of the independent variables while controlling for sociodemographic characteristics. Statistical significance was established at a *p*-value of less than 0.05.

## Results

### Descriptive Characteristics of the Study Participants

Table 1 presents the descriptive characteristics of study participants, both overall and comparing those who reported loneliness with those who did not across various primary independent and control variables. Demographically, participants were fairly evenly distributed across age groups, with the largest group being 35–44 years old (32.1%). The majority of participants were male (59.2%), married or in a relationship (63.8%), and college graduates (29.0%). A significant portion (69.2%) was currently employed, with the largest community represented in this study being Congolese (27.7%).

**Table 1:** Descriptive Characteristics of the Study Participants by Loneliness Status

Variable	Overall (N = 458; 100.0%)	Loneliness Status		
		No (N = 325; 71.0%)	Yes (N = 133; 29.0%)	*p*-value
*Primary Independent Variables*				
Access to Healthcare When Needed				0.270
Yes	353 (77.1)	255 (78.5)	98 (73.7)	
No	105 (22.9)	70 (21.5)	35 (26.3)	
Access to a Regular Doctor				< 0.001***
Yes	258 (56.3)	203 (62.5)	55 (41.4)	
No	200 (43.7)	122 (37.5)	78 (58.6)	
Self-Rated Health				0.037*
Excellent	75 (16.4)	58 (17.8)	17 (12.8)	
Very Good	139 (30.3)	106 (32.6)	33 (24.8)	
Good	170 (37.1)	117 (36.0)	53 (39.8)	
Fair/Poor	74 (16.2)	44 (13.5)	30 (22.6)	
Chronic Disease Status				0.098^¥^
None	328 (71.6)	240 (73.8)	88 (66.2)	
Single or Multiple Conditions	130 (28.4)	85 (26.2)	45 (33.8)	
Anxiety				< 0.001***
No	400 (87.3)	304 (93.5)	96 (72.2)	
Yes	58 (12.7)	21 (6.5)	37 (27.8)	
Depression				< 0.001***
No	395 (86.2)	294 (90.5)	101 (75.9)	
Yes	63 (13.8)	31 (9.5)	32 (24.1)	
Perceived Discrimination for Race				0.037
No	305 (66.6)	226 (69.5)	79 (59.4)	
Yes	153 (33.4)	99 (30.5)	54 (40.6)	
Resilience				< 0.001***
High	155 (33.8)	133 (40.9)	22 (16.5)	
Medium	158 (34.5)	114 (35.1)	44 (33.1)	
Low	145 (31.7)	78 (24.0)	67 (50.4)	
Social Support				< 0.001***
High	285 (62.2)	231 (71.1)	54 (40.6)	
Medium	147 (32.1)	79 (24.3)	68 (51.1)	
Low	26 (5.7)	15 (4.6)	11 (8.3)	
*Controls*				
Age (years)				0.023*
18–24	105 (22.9)	77 (23.7)	28 (21.1)	
25–34	111 (24.2)	82 (25.2)	29 (21.8)	
35–44	147 (32.1)	91 (28.0)	56 (42.1)	
45 +	95 (20.7)	75 (23.1)	20 (15.0)	
Sex at Birth				0.187
Male	271 (59.2)	186 (57.2)	85 (63.9)	
Female	187 (40.8)	139 (42.8)	48 (36.1)	
Marital Status				0.796
Married or In-a-Relationship	292 (63.8)	206 (63.4)	86 (64.7)	
Never Married/Separated/Divorced/Widowed	166 (36.2)	119 (36.6)	47 (35.3)	
Education				0.377
College Graduate	133 (29.0)	98 (30.2)	35 (26.3)	
Some College	86 (18.8)	63 (19.4)	23 (17.3)	
High School Graduate or Equivalent	120 (26.2)	87 (26.8)	33 (24.8)	
None or Less Than High School	119 (26.0)	77 (23.7)	42 (31.6)	
Current Employment Status				0.496
Employed	317 (69.2)	228 (70.2)	89 (66.9)	
Not Employed	141 (30.8)	97 (29.8)	44 (33.1)	
Community Represented				0.190
Afghan	95 (20.7)	66 (20.3)	29 (21.8)	
Bhutanese	81 (17.7)	64 (19.7)	17 (12.8)	
Congolese	127 (27.7)	84 (25.8)	43 (32.3)	
Ethiopian and/or Eritrean	81 (17.7)	54 (16.6)	27 (20.3)	
Somali	74 (16.2)	57 (17.5)	17 (12.8)	

All bivariate relationships were assessed using the Pearson’s Chi-square test. Significant p-values are bolded. *p < 0.05, **p < 0.01, ***p < 0.001. ^¥^approached significance

Over one-fifth (22.9%) of the participants could not access healthcare when needed, and about 44% did not have access to a regular doctor. In terms of health, 16.2% of respondents self-rated their health as fair or poor, 28.4% had one or more chronic conditions, 12.7% had anxiety, and 13.8% had depression. Over half (62.2%) reported high social support, with only 5.7% having low social support. One-third (33.4%) of the participants perceived discrimination based on race.

Regarding loneliness, 29% of participants reported feeling lonely, while 71% did not. The bivariate chi-square analysis revealed several factors significantly associated with loneliness. Participants with regular access to a doctor were less likely to report loneliness (41.4%) compared to those without access (58.6%, *p* < 0.001). Additionally, poor self-rated health, higher rates of anxiety and depression, lower resilience, and reduced social support were significantly more common among lonely participants.

The age distribution indicated that loneliness was most prevalent among participants aged 35–44 years (42.1%). Although sex at birth, marital status, education, employment status, and community representation showed varying percentages across loneliness groups, these differences did not reach statistical significance (Table 1).

### Loneliness Across Five Refugee Communities

Supplementary Table 1 provides a bivariate analysis of factors associated with loneliness across the five communities, revealing distinct patterns in healthcare access, physical and mental health, resilience, and social support. Afghan participants with limited access to a regular doctor were more likely to report loneliness (79.3% vs. 36.4%, *p* < 0.001), a trend also evident among Bhutanese refugees (52.9% vs. 17.2%, *p* = 0.002). Chronic health conditions were more closely tied to loneliness among Bhutanese participants (58.8% with conditions vs. 32.8% without, *p* = 0.049). Mental health challenges, especially anxiety and depression, showed strong associations with loneliness in Afghans (31.0% for anxiety, *p* < 0.001; 27.6% for depression, *p* = 0.037), Bhutanese (47.1% for anxiety, *p* < 0.001; 35.3% for depression, *p* = 0.027), and Somali participants (41.2% for anxiety, *p* = 0.002). Perceived racial discrimination was significantly linked to loneliness among Congolese participants (48.8% with discrimination vs. 28.6% without, *p* = 0.024). Resilience also played a role, with lower resilience levels associated with higher loneliness in Afghans (69.0% for low resilience, *p* < 0.001), Bhutanese (58.8%, *p* = 0.002), and Ethiopian/Eritrean refugees (51.9%, *p* = 0.011). Social support emerged as a critical factor across all communities, with low social support showing a strong correlation with loneliness. However, this association was statistically significant only among Afghan (*p* < 0.001), Bhutanese (*p* < 0.001), and Ethiopian/Eritrean communities (*p* = 0.006). Due to small sample sizes, subgroup regression analyses were not feasible; thus, only bivariate associations between key factors and loneliness across communities are presented (Supplementary Table 1).

### Factors Associated with Loneliness

Table 2 presents the unadjusted and adjusted odds ratios with 95% confidence intervals (CI), comparing the odds of experiencing loneliness across different levels of various primary independent variables, both before and after adjusting for potential confounders. The adjusted analyses highlighted the importance of access to a regular doctor, mental health status, resilience, and social support as key determinants of loneliness.

**Table 2:** Binary Logistic Regression for the Correlates of Loneliness Status

Primary Independent Variables	Unadjusted Odds Ratio (95%CI)	Adjusted Odds Ratio (95%CI)
**Access to Healthcare When Needed (ref = “No”)**		
Yes	0.77 (0.48–1.23)	0.76 (0.46–1.25)
**Access to a Regular Doctor (ref = “No”)**		
Yes	0.42 (0.28–0.64) ^***^	0.44 (0.29–0.69)^***^
**Self-Rated Health (ref = “Excellent”)**		
Very Good	1.06 (0.55–2.07)	1.09 (0.55–2.18)
Good	1.55 (0.82–2.90)	1.78 (0.92–3.43)^¥^
Fair/Poor	2.33 (1.14–4.74)^*^	3.07 (1.42–6.63)^**^
**Chronic Disease Status (ref = “None”)**		
Single or Multiple Conditions	1.44 (0.93–2.23)^¥^	1.66 (1.03–2.68)^*^
**Anxiety (ref = “No”)**		
Yes	5.58 (3.12–9.99)^***^	8.43 (4.46–15.93)^***^
**Depression (ref = “No”)**		
Yes	3.01 (1.75–5.17)^***^	3.53 (1.99–6.26)^***^
**Perceived Discrimination for Race (ref = “No”)**		
Yes	1.56 (1.03–2.37)^*^	1.73 (1.10–2.74)^*^
**Resilience (ref = “High”)**		
Medium	2.33 (1.32–4.12)^**^	2.41 (1.34–4.36)^**^
Low	5.19 (2.98–9.06)^***^	5.07 (2.79–9.20)^***^
**Social Support (ref = “High”)**		
Medium	3.68 (2.37–5.71)^***^	3.39 (2.15–5.36)^***^
Low	3.13 (1.36–7.21)^**^	2.50 (1.04–6.03)^*^

Adjusted model controlled for sociodemographic variables that included age, sex, marital status, education, current employment, and community represented. Abbreviation: CI: Confidence Interval. ^*^p < 0.05, ^**^p < 0.01, ^***^p < 0.001, ^¥^approached significance

Participants with regular access to a doctor were 56% less likely to experience loneliness than those without access, after controlling for all other sociodemographic variables in the model [adjusted odds ratio (aOR) = 0.44, 95% CI: 0.29–0.69]. As self-rated health declined, the odds of loneliness increased significantly. Participants who rated their health as fair or poor were over three times more likely to be lonely compared to those with excellent health (aOR = 3.07, 95% CI: 1.42–6.63). Having one or more chronic conditions was linked to higher odds of loneliness, with participants facing about 1.7 times greater odds of loneliness compared to those without chronic conditions (aOR = 1.66, 95% CI: 1.03–2.68). Both anxiety and depression were strongly associated with loneliness. Participants with anxiety had more than eight times the odds of experiencing loneliness (aOR = 8.43, 95% CI: 4.46–15.93), while those with depression were over three times as likely to be lonely (aOR = 3.53, 95% CI: 1.99–6.26).

Participants who reported experiencing racial discrimination were 73% more likely to report loneliness compared to those who did not experience racial discrimination (aOR = 1.73, 95% CI: 1.10–2.74). Lower resilience and social support were strongly linked to increased odds of loneliness. Participants with medium or low resilience had significantly higher odds of loneliness than those with high resilience (aOR = 2.41, 95% CI: 1.34–4.36; aOR = 5.07, 95% CI: 2.79–9.20, respectively). Similarly, those with medium or low social support had significantly higher odds of loneliness compared to those with high social support (aOR = 3.39, 95% CI: 2.15–5.36; aOR = 2.50, 95% CI: 1.04–6.03, respectively).

## Discussion

This study assessed the prevalence of loneliness and its associated factors among adults from five different resettled communities in Ohio. The findings revealed a high prevalence of loneliness within these populations, which was significantly associated with several health-related factors, including self-reported health status, chronic illness, anxiety, depression, and access to a regular healthcare provider. Additionally, psychosocial variables, such as perceived racial discrimination, resilience, and social support, were found to be strongly linked to loneliness in this population.

### High Prevalence of Loneliness Among Resettled Communities

One noteworthy finding was that one-third (29.0%) of the participants in this study reported feelings of loneliness. This finding is consistent with previous research indicating that resettled populations are at a higher risk of experiencing loneliness compared to host populations [9]. A recent systematic review found that the prevalence of loneliness among refugees resettled in high-income countries ranged from 15.9% to 47.7% [9]. The prevalence of loneliness estimated in our study falls within this range, albeit on the lower end, yet it still emphasizes a significant public health issue that requires attention. It is important to note that the prevalence of loneliness in our study may be underestimated due to certain methodological limitations—most notably, the use of an English-only online survey. The limitations, discussed in detail below under study limitations, suggest that the true extent of loneliness in these communities could be even higher than reported. This increased vulnerability to loneliness among resettled adults may be attributed to several factors unique to their experiences, including forced displacement, the loss of social networks, language barriers, and cultural dislocation [10, 43]. They often experience social isolation as they attempt to navigate unfamiliar environments, cope with trauma, and rebuild their lives in a new country, which makes them more susceptible to loneliness.

### Mental Health and Loneliness

Mental health stood out as the strongest associated factor. Participants experiencing anxiety were over eight times more likely to report loneliness, while those with depression were more than three times as likely to feel lonely. These findings are consistent with previous research showing a close link between poor mental health and social isolation [9, 44]. Refugees are especially vulnerable to psychological distress due to pre-migration trauma and post-resettlement challenges [45]. Studies show that nearly one in three refugees in high-income countries experience severe depression, anxiety, or PTSD [46]; rates significantly higher than those of host populations [47], with some reports indicating rates up to seven to eight times higher among Syrian refugees than those in the general population of their host countries [48].

Individuals suffering from anxiety and depression often experience social withdrawal, feelings of fear, low self-esteem, and a lack of energy, which can make it difficult for them to seek or maintain relationships, further isolating them from essential support networks [48, 49]. Importantly, the connection between mental health disorders and loneliness is likely bidirectional. On the one hand, mental health challenges lead to loneliness, but on the other hand, loneliness itself can exacerbate existing mental health issues. Research indicates that prolonged loneliness triggers physiological stress responses that disrupt sleep, alter brain function, and increase vulnerability to, as well as exacerbate, symptoms of depression and anxiety [50]. Consequently, loneliness becomes both a symptom and a consequence of mental health struggles, creating a vicious cycle in which isolation worsens mental health, and deteriorating mental health intensifies loneliness [51].

### Physical Health and Loneliness

Concerning physical health, chronic conditions and self-reported poor health emerged as significant factors associated with loneliness in this study. Chronic conditions and self-reported poor health can greatly exacerbate feelings of loneliness, a finding consistent with previous research [9]. Among the resettled population, chronic illnesses often accumulate over time due to prolonged exposure to adverse conditions, such as food insecurity, limited access to healthcare, and mental health challenges both in refugee camps and after resettlement in host countries [45, 52]. These chronic and cumulative health issues can severely restrict physical mobility and reduce participation in social activities, further deepening the sense of isolation and disconnection from their new communities [53]. Additionally, the stigma surrounding illness and the perception of poor health, particularly in an unfamiliar environment without established social support networks, can further intensify feelings of loneliness [54]. Refugees may withdraw from social interactions due to embarrassment, fear of judgment, or physical limitations related to their health conditions. This can create a self-reinforcing cycle in which loneliness worsens physical and mental health, and declining health further deepens social isolation [44, 53, 55].

### Healthcare Access and Loneliness

Having regular access to a healthcare provider was found to be protective against loneliness in this study, with participants who had a doctor for regular check-ups being less likely to report feelings of loneliness. This finding is particularly interesting, as another variable—general access to healthcare when needed—was associated with lower odds of loneliness but was not statistically significant. The key difference may lie in the continuity of care—ongoing, relationship-based interactions with a consistent provider—and episodic care, which occurs only when immediate medical attention is required. Regularly seeing a familiar face can foster a sense of personal connection, trust, security, and stability, which may alleviate feelings of isolation [56]. For many refugees, the healthcare provider becomes a consistent figure in an otherwise unfamiliar environment, offering not only medical support but also an opportunity for social interaction and emotional reassurance. This regular contact can build a sense of belonging and connection, reducing the psychological burden of loneliness. Furthermore, the routine of attending regular check-ups may serve as a marker of broader social integration [56]. Refugees with established healthcare routines may also have better access to other social services and support networks, including referrals to mental health services, health education, and community-based resources, further enhancing their sense of inclusion and buffering against loneliness [57, 58]. In contrast, episodic care—such as walk-in visits or emergency care—may address immediate health needs but lacks the relational depth or predictability needed to build rapport.

### Racial Discrimination and Loneliness

Racial discrimination was another significant factor, associated with a 73% increase in the odds of experiencing loneliness, highlighting the profound influence of external social and environmental factors on refugees'sense of belonging. This finding aligns with broader literature that demonstrates the detrimental effects of discrimination on both social and psychological well-being [9]. Experiences of racism and discrimination often result in social exclusion, marginalization, and a diminished sense of integration into the host society, all of which significantly contribute to heightened feelings of loneliness [9, 43]. Racial discrimination disrupts social connections by fostering a sense of"otherness,"making refugees feel unwelcome and excluded from the broader community. This exclusion can limit their ability to form meaningful relationships, both within and outside their own ethnic groups, further deepening isolation. The emotional toll of repeated discrimination can also lead to withdrawal from social interactions, as individuals may fear encountering prejudice or hostility. Over time, this erodes their confidence in seeking social support and participating in community activities, reinforcing feelings of loneliness [43]. Additionally, racial discrimination creates structural barriers that hinder access to essential resources such as education, employment, and healthcare, further isolating them from mainstream society [43]. This multifaceted impact of racial discrimination—social, emotional, and structural—plays a central role in increasing loneliness among resettled populations.

### Resilience, Social Support and Loneliness

Protective factors such as social support and resilience were significant determinants of loneliness in this study. Higher levels of both were significantly associated with lower odds of experiencing loneliness. These findings align with existing literature, which suggests that strong social support [9] and resilience [59, 60] are linked to reduced loneliness. This underscores the crucial role that resilience, social networks, and relationships play in mitigating isolation [9, 59, 60].

Several mechanisms may help to explain the relationship between social support and loneliness. Social support, whether emotional, instrumental, or informational, provides resettled individuals with a sense of connection and belonging, which helps counter feelings of isolation [61]. Supportive relationships with family and friends also act as a buffer against stress, offering practical help in navigating challenges such as language barriers, cultural adaptation, and access to resources [38]. These help to foster a sense of security and inclusion, thereby contributing to diminished risk of loneliness. In contrast, a lack of social support leaves them vulnerable to isolation, potentially because of fewer opportunities to interact, share experiences, or receive help from others. Despite the buffering role of social support in promoting well-being, many resettled people are still vulnerable to physical separation from family, friends, and social networks in their country of origin and consequently often struggle to establish new connections due to language barriers, cultural differences, and the challenges of adapting to a new social environment [9, 60]. Fortunately, social support can be strengthened through culturally tailored activities that foster social connectedness [62]. These activities might include community programs that celebrate shared cultural traditions, language exchange initiatives, and mentorship programs that pair resettled families with local individuals or families.

It is important to highlight resilience, which refers to an individual’s capacity to adapt and recover from adversity [63]. Resilient individuals are often better equipped to cope with life's challenges and stressors, including social isolation, and are more adaptable to change [60, 64], making them less vulnerable to the emotional strain of loneliness. Previous research has shown that psychological resilience serves as a crucial resource for refugees, enabling them to navigate uncertainty, trauma, and the stress of adapting to a new life while remaining open to social connections [59]. Such mental strength allows resilient individuals to seek out and maintain relationships, even when facing obstacles, thereby shielding them from the isolation that could have resulted from disconnection [59, 63, 64]. In contrast, those with low resilience may struggle to recover from setbacks, leading to a cycle of emotional withdrawal and heightened loneliness. Together, social support and resilience create a protective framework that enhances one's ability to engage with their communities and build meaningful social ties, which are crucial in reducing the experience of loneliness.

### Community-Specific Variations in Loneliness Experiences

Subgroup analyses revealed important variations across ethnic groups that warrant attention. For example, loneliness was significantly associated with lack of access to a regular doctor among Afghan and Bhutanese participants, while perceived racial discrimination emerged as a significant factor among Congolese respondents. Mental health challenges showed a significant association with loneliness in all targeted communities. Low resilience was significantly linked to higher loneliness in all subgroups except Somali participants. Similarly, low social support was significantly associated with loneliness in all subgroups except the Congolese and Somali participants. These patterns suggest that interventions must be tailored not only to cultural background but also to the specific needs and experiences of each community. However, small sample sizes within subgroups may have contributed to non-significant findings in some cases, despite observable trends—potentially obscuring meaningful associations. Additionally, the limited sample size prevented us from conducting adjusted logistic regression analyses by community, restricting our ability to control for potential confounding factors. As such, while the bivariate differences provide useful direction, they should be interpreted with caution and not be overextended to drive definitive programmatic recommendations. Future research should prioritize larger, community-stratified samples and employ multivariate approaches to generate more robust, nuanced findings that can inform targeted interventions and culturally responsive policies.

### Strengths, Limitations, and Future Research Directions

This study offers several important contributions. A key strength is that the study successfully engaged a large sample of hard-to-reach groups, coupled with the inclusion of multi-ethnic resettled communities, and has practical relevance and timely application to real-world challenges faced by these communities, often underrepresented in population-level public health research. By identifying distinct risk and protective factors for loneliness across diverse refugee groups in a non-traditional resettlement state like Ohio, the study provides actionable insights for practitioners, policymakers, and community-based organizations seeking to address social and mental health disparities among resettled populations. Another significant strength is the engagement of local community leaders and faith-based institutions during various phases of the research study strengthened the study's design and tool development, ensuring the content's appropriateness.

A significant limitation of the study is its cross-sectional design, which limits the ability to determine causal relationships between key variables such as healthcare access, mental health, and loneliness. While the study identifies important statistical associations, the direction of these relationships remains unclear due to cross-sectional data. Additionally, the use of an English-only, online survey likely excluded key subpopulations within resettled communities—particularly older adults, individuals with limited English proficiency, those with low literacy, and persons with limited access to digital devices or internet connectivity. These groups are often more socially and economically marginalized, and thus may be at higher risk for loneliness. Their exclusion may have skewed the findings toward a more acculturated and digitally connected segment of the population, potentially leading to an underestimation of loneliness prevalence. An additional limitation in terms of measurement bias is also anticipated. Specifically, access to healthcare and access to a regular doctor were assessed using single-item questions, chosen for their simplicity, clarity, and cultural accessibility, which helped minimize respondent burden in a linguistically and culturally diverse population. This approach aimed to ensure that participants from varying literacy levels and language backgrounds could engage meaningfully with the survey content. However, the use of single-item measures may limit the precision of the constructs assessed and introduce potential measurement bias. Another limitation related to measurement is the reliance on self-reported refugee status for eligibility, which may introduce misclassification—particularly due to social desirability bias—given the stigma and prejudice often associated with refugee populations.

The narrow geographic scope of this study presents a limitation to generalizability, as most participants were recruited from the central Columbus area through a local refugee-serving organization. The urban and suburban study settings, characterized by relatively strong infrastructure, multilingual services, and established cultural organizations, may have influenced participants’ experiences of loneliness. Refugees in rural or underserved areas often face distinct challenges, such as reduced access to services, lower cultural diversity, and limited social support, which could elevate the experience of loneliness. As a result, our findings may be underestimated, and caution is warranted when generalizing these results to other parts of Ohio or to different states. While some cities or towns may be less welcoming or accommodating to resettled communities, the influence of geographic context may be modest, as the majority of resettled refugees in the U.S. live in urban or suburban areas, with only 14% residing in rural locations [65]. Moreover, a prior study found no significant differences in depression prevalence across four Ohio cities, suggesting that mental health outcomes may be relatively stable across similar urban contexts [66].

Future research may focus on using longitudinal designs to clarify the direction of the relationships observed in this study over time and to broaden the scope for a better comprehension of how pre-migration experiences and post-resettlement challenges affect resettled populations. Additionally, employing mixed-method approaches that integrate quantitative data with qualitative insights may provide a more nuanced understanding of resettled adults’ experiences pre- and post-resettlement, to facilitate the development of more effective and comprehensive programs and policies. To overcome the limitations related to language and technology and to foster broader participation, future studies should be geared towards offering surveys in multiple languages and providing both paper-based and online formats.

Given the strong association between anxiety and loneliness observed in this study, future work could explore whether early intervention for anxiety symptoms among resettled refugees helps prevent the onset or intensification of loneliness over time. Moreover, the strong protective effect of having a regular doctor underscores the importance of continuity of care. Future research should explore how consistent provider relationships foster social connectedness and mitigate loneliness, especially in comparison to more fragmented, episodic healthcare use. Implementation research is needed to evaluate culturally grounded programs that aim to strengthen resilience and social support networks.

## Conclusions and Implications

This study highlighted the prevalence and factors contributing to loneliness among multi-ethnic resettled communities in Ohio, revealing the complex interplay of health, social, and psychological elements. Nearly one-third of the surveyed participants reported experiencing loneliness, highlighting the significant magnitude of the issue. Key factors associated with loneliness include access to a regular doctor, mental health challenges, self-rated health, chronic disease, racial discrimination, and insufficient social support. These findings underscore the urgent need among resettled refugee communities for culturally tailored interventions that enhance healthcare access, integrate mental health services, address discrimination, and strengthen resilience and social support. Such efforts are essential not only for improving individual well-being but also for building stronger, more inclusive, and resilient communities.

The study has several important implications for practice and policy. First and foremost, the study findings emphasize the urgent need for comprehensive public health strategies and programmatic interventions that address the intertwined mental, physical, and social needs of resettled adults. Policymakers and community organizations must prioritize improving healthcare access, providing robust mental health services, and fostering stronger social connections to effectively combat loneliness in these vulnerable populations. An integrated, multifaceted, and innovative approach is crucial, utilizing mobile health clinics, telehealth services, and community health worker programs to bridge the socio-cultural and financial barriers that often prevent them from accessing healthcare.

Second, healthcare providers play a pivotal role and should routinely screen for loneliness during medical visits. Incorporating loneliness assessments into primary care would allow for early identification of at-risk individuals and provide them with timely resources and interventions. Routine screening is critical for refugee populations due to their tendency toward somatization, where distress manifests as physical symptoms, often masking underlying psychological conditions that require timely intervention [67]. In parallel, targeted programs focusing on health education, preventive screenings, and the management of chronic diseases and mental health issues can significantly enhance overall well-being while mitigating the factors that contribute to loneliness.

Third, given the study finding of mental health challenges being significantly associated with loneliness in all targeted communities, mental health services, including counseling, therapy, and stress management initiatives, are essential to improving mental resilience and reducing the emotional toll of isolation. The clear benefit of having a regular doctor suggests that policies promoting continuity of care may reduce loneliness and improve overall well-being. Interventions such as community health worker programs or refugee-centered patient navigation services could help foster consistent, trusting relationships within the healthcare system. One previous research has suggested that interventions to lessen the risks of mental ill-health can start with the prevention of social isolation [68].

Fourth, given the finding that low social support was significantly associated with loneliness in three (Afghan, Bhutanese, and Ethiopian/Eritrean) of the five subgroups, culturally innovative programs to enhance social support and to promote social support groups may have a beneficial impact in reducing and enhancing illness for resettled communities. It may be relevant to draw insights from a ‘systems thinking’ guided research that developed a theory regarding the causes of loneliness and the functioning of interventions among migrant and ethnic minority populations. The study found strongest evidence of positive impact on loneliness among shared-identity social support groups [15].

Last, community-based initiatives are also vital in addressing loneliness, as they promote social engagement and create supportive environments for the resettled population. Expanding targeted programs, specifically in refugee resettlement areas, such as support groups, community centers, and organized social activities can help individuals build meaningful connections, fostering a sense of belonging and reducing feelings of loneliness. Given the diverse cultural backgrounds of resettled communities, all interventions must be culturally tailored to respect and address the unique experiences and needs of each community. Moreover, the protective role of resilience against loneliness suggests that programs aimed at building personal resilience—such as stress management workshops, coping skills training, and peer support groups—could be highly beneficial. Research interestingly points to common conceptualizations of ‘loneliness’ that can be beneficially utilized to recognize four proximate determinants when focusing on migrant and ethnic minority populations: positive social ties and interactions, negative social ties and interactions, self-worth, and appraisal of existing ties [15]. These efforts not only help individuals manage the stresses of resettlement but also empower them to cultivate lasting social bonds, improving both their mental and social well-being.

## Supplementary Information

Below is the link to the electronic supplementary material.

ESM 1 (DOCX 29.4 KB)

## Data Availability

The datasets generated and/or analyzed during this study are not publicly available due to privacy and confidentiality concerns. They contain sensitive information regarding individuals'demographics, experiences, and physical and mental health, which must be protected to safeguard participant privacy and dignity. However, de-identified datasets can be made available upon reasonable request to the corresponding author, contingent on obtaining the necessary ethical approval.
