# Supplementary material for: Loneliness in Resettlement Among Multi-Ethnic Resettled Refugees in Ohio
Source: J Racial Ethn Health Disparities. 2025 Sep 30;13(6):4674–90. doi: 10.1007/s40615-025-02614-3 (PMC13754950; doi:10.1007/s40615-025-02614-3)
Supplement: Supplementary file 1 [file 40615_2025_2614_MOESM1_ESM.docx]

**Supplementary Table 1. Bivariate Association between Primary Independent Variables and Loneliness Status Across Five Communities**

|  | **Afghan** | | | | **Bhutanese Nepali** | | | | **Congolese** | | | | **Ethiopian and/or Eritrean** | | | | **Somali** | | | |
| --- | --- | --- | --- | --- | --- | --- | --- | --- | --- | --- | --- | --- | --- | --- | --- | --- | --- | --- | --- | --- |
|  | **Overall n (%)** | **Not Lonely n (%)** | **Lonely n (%)** | **p-Value** | **Overall n (%)** | **Not Lonely n (%)** | **Lonely n (%)** | **p-Value** | **Overall n (%)** | **Not Lonely n (%)** | **Lonely n (%)** | **p-Value** | **Overall n (%)** | **Not Lonely n (%)** | **Lonely n (%)** | **p-Value** | **Overall n (%)** | **Not Lonely n (%)** | **Lonely n (%)** | **p-Value** |
| **Healthcare**  **access** |  |  |  |  |  |  |  |  |  |  |  |  |  |  |  |  |  |  |  |  |
| Yes | 26 (27.4) | 17 (25.8) | 9 (31.0) | 0.5952 | 19 (23.5) | 14 (21.9) | 5 (29.4) | 0.5145 | 26 (20.5) | 17 (20.2) | 9 (20.9) | 0.9271 | 12 (14.8) | 5 (9.3) | 7 (25.9) | **0.0465** | 22 (29.7) | 17 (29.8) | 5 (29.4) | 0.9739 |
| No | 69 (72.6) | 49 (74.2) | 20 (69.0) |  | 62 (76.5) | 50 (78.1) | 12 (70.6) |  | 101 (79.5) | 67 (79.8) | 34 (79.1) |  | 69 (85.2) | 49 (90.7) | 20 (74.1) |  | 52 (70.3) | 40 (70.2) | 12 (70.6) |  |
| **Discrimination by race** |  |  |  |  |  |  |  |  |  |  |  |  |  |  |  |  |  |  |  |  |
| No | 79 (83.2) | 56 (84.8) | 23 (79.3) | 0.5065 | 51 (63.0) | 41 (64.1) | 10 (58.8) | 0.6909 | 82 (64.6) | 60 (71.4) | 22 (51.2) | **0.0238** | 53 (65.4) | 35 (64.8) | 18 (66.7) | 0.8688 | 40 (54.1) | 34 (59.6) | 6 (35.3) | 0.077 |
| Yes | 16 (16.8) | 10 (15.2) | 6 (20.7) |  | 30 (37.0) | 23 (35.9) | 7 (41.2) |  | 45 (35.4) | 24 (28.6) | 21 (48.8) |  | 28 (34.6) | 19 (35.2) | 9 (33.3) |  | 34 (45.9) | 23 (40.4) | 11 (64.7) |  |
| **Access to**  **doctor** |  |  |  |  |  |  |  |  |  |  |  |  |  |  |  |  |  |  |  |  |
| No | 47 (49.5) | 24 (36.4) | 23 (79.3) | **0.0001** | 20 (24.7) | 11 (17.2) | 9 (52.9) | **0.0024** | 65 (51.2) | 41 (48.8) | 24 (55.8) | 0.4549 | 27 (33.3) | 15 (27.8) | 12 (44.4) | 0.1336 | 41 (55.4) | 31 (54.4) | 10 (58.8) | 0.7467 |
| Yes | 48 (50.5) | 42 (63.6) | 6 (20.7) |  | 61 (75.3) | 53 (82.8) | 8 (47.1) |  | 62 (48.8) | 43 (51.2) | 19 (44.2) |  | 54 (66.7) | 39 (72.2) | 15 (55.6) |  | 33 (44.6) | 26 (45.6) | 7 (41.2) |  |
| **Self-rated health** |  |  |  |  |  |  |  |  |  |  |  |  |  |  |  |  |  |  |  |  |
| Fair/Poor | 10 (10.5) | 6 (9.1) | 4 (13.8) | 0.1741 | 20 (24.7) | 14 (21.9) | 6 (35.3) | 0.1741 | 17 (13.4) | 10 (11.9) | 7 (16.3) | 0.1728 | 13 (16.0) | 6 (11.1) | 7 (25.9) | 0.3832 | 14 (18.9) | 8 (14.0) | 6 (35.3) | 0.3832 |
| Good | 30 (31.6) | 18 (27.3) | 12 (41.4) |  | 34 (42.0) | 26 (40.6) | 8 (47.1) |  | 48 (37.8) | 33 (39.3) | 15 (34.9) |  | 25 (30.9) | 17 (31.5) | 8 (29.6) |  | 33 (44.6) | 23 (40.4) | 10 (58.8) |  |
| Very Good | 39 (41.1) | 32 (48.5) | 7 (24.1) |  | 21 (25.9) | 21 (32.8) | 0 (.) |  | 39 (30.7) | 22 (26.2) | 17 (39.5) |  | 29 (35.8) | 21 (38.9) | 8 (29.6) |  | 11 (14.9) | 10 (17.5) | 1 (5.9) |  |
| Excellent | 16 (16.8) | 10 (15.2) | 6 (20.7) |  | 6 (7.4) | 3 (4.7) | 3 (17.6) |  | 23 (18.1) | 19 (22.6) | 4 (9.3) |  | 14 (17.3) | 10 (18.5) | 4 (14.8) |  | 16 (21.6) | 16 (28.1) | 0 (.) |  |
| **Disease status** |  |  |  |  |  |  |  |  |  |  |  |  |  |  |  |  |  |  |  |  |
| Without disease | 77 (81.1) | 54 (81.8) | 23 (79.3) | 0.7739 | 50 (61.7) | 43 (67.2) | 7 (41.2) | **0.0498** | 88 (69.3) | 63 (75.0) | 25 (58.1) | 0.0513 | 57 (70.4) | 39 (72.2) | 18 (66.7) | 0.6057 | 56 (75.7) | 41 (71.9) | 15 (88.2) | 0.1691 |
| With disease | 18 (18.9) | 12 (18.2) | 6 (20.7) |  | 31 (38.3) | 21 (32.8) | 10 (58.8) |  | 39 (30.7) | 21 (25.0) | 18 (41.9) |  | 24 (29.6) | 15 (27.8) | 9 (33.3) |  | 18 (24.3) | 16 (28.1) | 2 (11.8) |  |
| **Anxiety** |  |  |  |  |  |  |  |  |  |  |  |  |  |  |  |  |  |  |  |  |
| No | 83 (87.4) | 63 (95.5) | 20 (69.0) | **0.0003** | 68 (84.0) | 59 (92.2) | 9 (52.9) | **<.0001** | 115 (90.6) | 79 (94.0) | 36 (83.7) | 0.0597 | 72 (88.9) | 51 (94.4) | 21 (77.8) | **0.0244** | 62 (83.8) | 52 (91.2) | 10 (58.8) | **0.0015** |
| Yes | 12 (12.6) | 3 (4.5) | 9 (31.0) |  | 13 (16.0) | 5 (7.8) | 8 (47.1) |  | 12 (9.4) | 5 (6.0) | 7 (16.3) |  | 9 (11.1) | 3 (5.6) | 6 (22.2) |  | 12 (16.2) | 5 (8.8) | 7 (41.2) |  |
| **Depression** |  |  |  |  |  |  |  |  |  |  |  |  |  |  |  |  |  |  |  |  |
| No | 80 (84.2) | 59 (89.4) | 21 (72.4) | **0.0366** | 67 (82.7) | 56 (87.5) | 11 (64.7) | **0.0271** | 111 (87.4) | 77 (91.7) | 34 (79.1) | **0.0429** | 73 (90.1) | 51 (94.4) | 22 (81.5) | 0.0653 | 64 (86.5) | 51 (89.5) | 13 (76.5) | 0.1687 |
| Yes | 15 (15.8) | 7 (10.6) | 8 (27.6) |  | 14 (17.3) | 8 (12.5) | 6 (35.3) |  | 16 (12.6) | 7 (8.3) | 9 (20.9) |  | 8 (9.9) | 3 (5.6) | 5 (18.5) |  | 10 (13.5) | 6 (10.5) | 4 (23.5) |  |
| **Resilience** |  |  |  |  |  |  |  |  |  |  |  |  |  |  |  |  |  |  |  |  |
| Low | 38 (40.0) | 18 (27.3) | 20 (69.0) | **0.0004** | 21 (25.9) | 11 (17.2) | 10 (58.8) | **0.002** | 45 (35.4) | 29 (34.5) | 16 (37.2) | **0.0035** | 25 (30.9) | 11 (20.4) | 14 (51.9) | **0.0109** | 16 (21.6) | 9 (15.8) | 7 (41.2) | 0.0538 |
| Medium | 27 (28.4) | 21 (31.8) | 6 (20.7) |  | 33 (40.7) | 30 (46.9) | 3 (17.6) |  | 42 (33.1) | 21 (25.0) | 21 (48.8) |  | 24 (29.6) | 17 (31.5) | 7 (25.9) |  | 32 (43.2) | 25 (43.9) | 7 (41.2) |  |
| High | 30 (31.6) | 27 (40.9) | 3 (10.3) |  | 27 (33.3) | 23 (35.9) | 4 (23.5) |  | 40 (31.5) | 34 (40.5) | 6 (14.0) |  | 32 (39.5) | 26 (48.1) | 6 (22.2) |  | 26 (35.1) | 23 (40.4) | 3 (17.6) |  |
| **Social support** |  |  |  |  |  |  |  |  |  |  |  |  |  |  |  |  |  |  |  |  |
| Low | 6 (6.3) | 3 (4.5) | 3 (10.3) | **0.0091** | 2 (2.5) | 1 (1.6) | 1 (5.9) | **<.0001** | 8 (6.3) | 6 (7.1) | 2 (4.7) | 0.0698 | 6 (7.4) | 3 (5.6) | 3 (11.1) | **0.0062** | 4 (5.4) | 2 (3.5) | 2 (11.8) | 0.3663 |
| Medium | 34 (35.8) | 18 (27.3) | 16 (55.2) |  | 19 (23.5) | 8 (12.5) | 11 (64.7) |  | 42 (33.1) | 22 (26.2) | 20 (46.5) |  | 28 (34.6) | 13 (24.1) | 15 (55.6) |  | 24 (32.4) | 18 (31.6) | 6 (35.3) |  |
| High | 55 (57.9) | 45 (68.2) | 10 (34.5) |  | 60 (74.1) | 55 (85.9) | 5 (29.4) |  | 77 (60.6) | 56 (66.7) | 21 (48.8) |  | 47 (58.0) | 38 (70.4) | 9 (33.3) |  | 46 (62.2) | 37 (64.9) | 9 (52.9) |  |
